# Myhre Syndrome Presenting With Congenital Proximal Radioulnar Synostosis: A Case Report

**DOI:** 10.7759/cureus.102594

**Published:** 2026-01-29

**Authors:** I-Shou Lin, Hsiu-Jung Lee, Wei-Ning Chang

**Affiliations:** 1 Department of Pediatrics, Kaohsiung Veterans General Hospital, Kaohsiung, TWN; 2 Department of Pediatrics, Tainan Hospital, Ministry of Health and Welfare, Tainan, TWN; 3 Department of Orthopedics, Kaohsiung Veterans General Hospital, Kaohsiung, TWN

**Keywords:** case report, musculoskeletal abnormalities, myhre syndrome, proximal radioulnar synostosis, smad4

## Abstract

Myhre syndrome (MS) is a rare, autosomal dominant multisystem disorder. Clinical features include short stature, variable degrees of intellectual disability, distinctive facial dysmorphism, musculoskeletal abnormalities, cardiopulmonary disorders, and abnormal sexual development. We report on an 11-year-old male Taiwanese patient who was initially referred to our genetic counseling clinic due to congenital proximal radioulnar synostosis (PRUS) and clinical suspicion of mucopolysaccharidosis. A pathogenic heterozygous missense variant in *SMAD4*, c.1498A>G (p.Ile500Val), was subsequently identified, confirming the diagnosis of MS. This case demonstrates the typical clinical phenotype along with the unique finding of PRUS, which has not been previously reported in association with this syndrome. This report highlights PRUS as a rare skeletal manifestation, expanding the known clinical spectrum of MS and providing valuable insights for clinical recognition.

## Introduction

Myhre syndrome (MS; OMIM #139210) is a rare, autosomal dominant disorder first described by Myhre et al. [1] in 1981. MS is caused by heterozygous missense variants in the *SMAD4* gene [2]. The syndrome presents with a complex, multisystemic phenotype that includes short stature, intellectual disability, distinctive facial dysmorphism (e.g., midface hypoplasia, narrow mouth, and prognathism), brachydactyly, clinodactyly, hearing loss, decreased joint mobility, and progressive stiffness of skin and joints, alongside significant cardiopulmonary involvement [2,3]. Although musculoskeletal abnormalities are well-documented in MS, the occurrence of congenital proximal radioulnar synostosis (PRUS) remains an underexplored manifestation. Documenting such cases is crucial to broaden the understanding of the syndrome's diverse skeletal phenotypic spectrum.

## Case presentation

An 11-year-old male presented to the orthopedic clinic with chief complaints of markedly restricted motion in both forearms, limited elbow flexion, a tiptoe gait, and intellectual disability. Retrospective review of the history indicated that the patient had undergone surgery at age three at another medical center for congenital PRUS. Physical examination revealed bilateral elbow flexion limited to 90 degrees and fixed bilateral forearm rotation in 45 degrees of pronation. Contractures of the bilateral heel cords and rectus femoris muscles were noted. When standing, the patient exhibited forward flexion of the trunk; if attempting to straighten his back, he was forced to stand on his toes.

**Figure 1:**
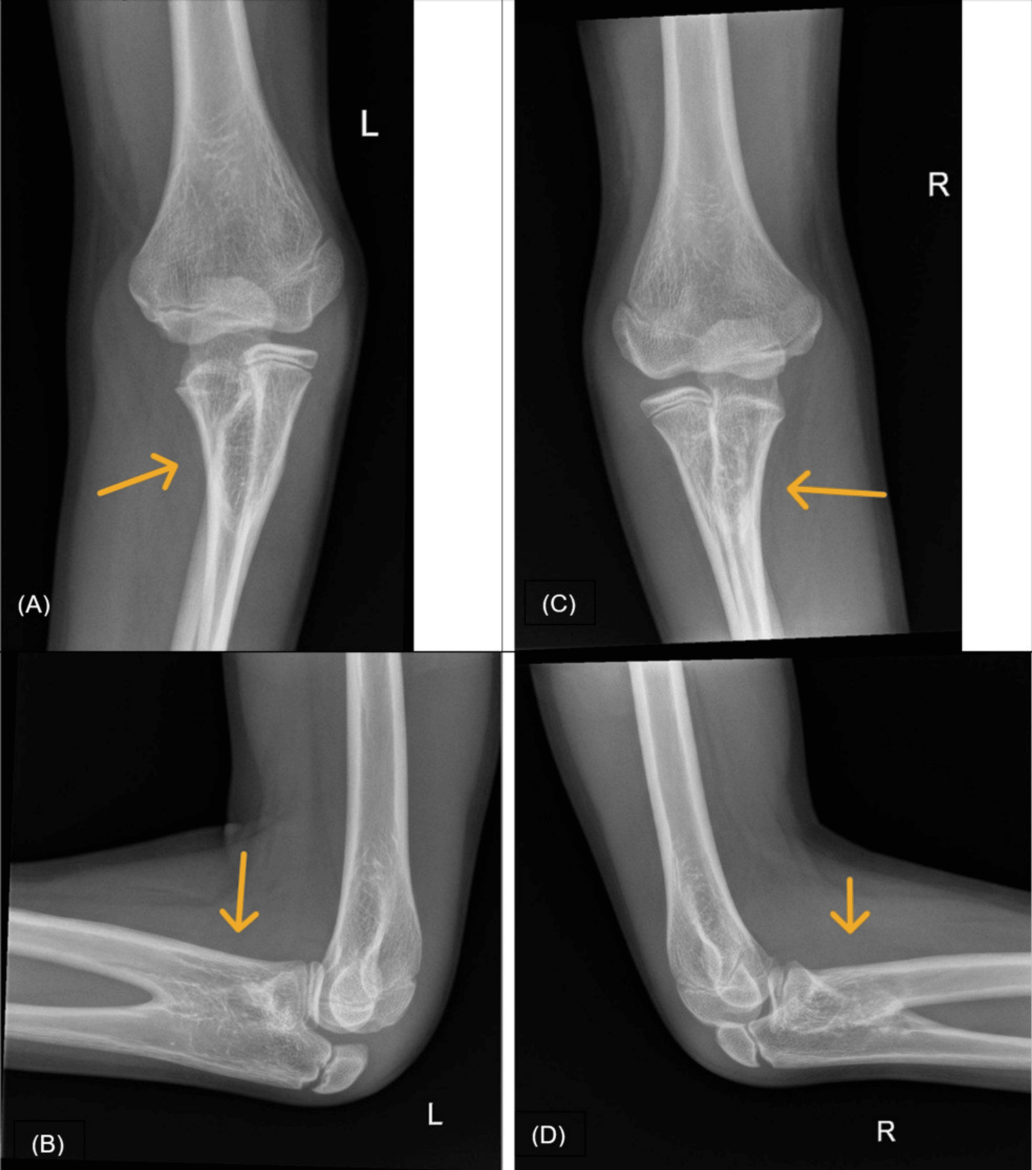
Radiographic findings of the bilateral elbows (A) AP and (B) lateral radiographs of the left elbow; (C) AP and (D) lateral radiographs of the right elbow. The images demonstrate bilateral PRUS, characterized by the osseous fusion of the proximal radius and ulna (indicated by arrows). AP, anteroposterior; PRUS, proximal radioulnar synostosis

**Figure 2:**
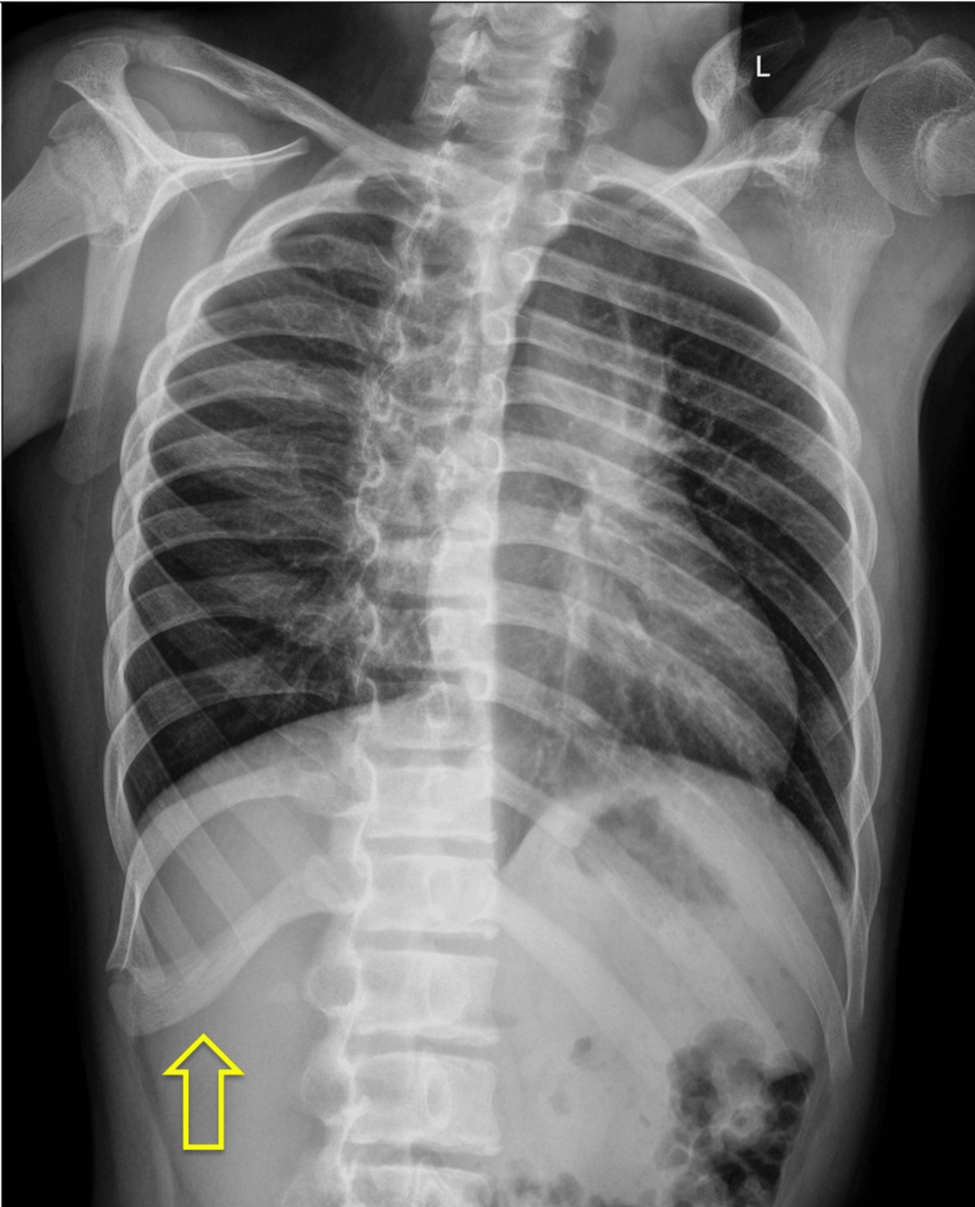
Chest radiograph The image demonstrates paddle-shaped widening of the ribs (arrow).

Orthopedic radiographs confirmed the previously known PRUS (Figure 1A-1D) and revealed paddle-shaped widened ribs on the chest radiograph (Figure 2). Given the combination of clinical and radiological findings, mucopolysaccharidosis (MPS) was initially suspected, leading to a referral to the genetic counseling clinic.

Upon referral, the patient’s birth history was noted as G1P1. Prenatal checkups were unremarkable. The patient was delivered at a gestational age of 36+4 weeks with a birth weight of 2600 g. The past medical history was unremarkable, with no major systemic diseases, no history of long-term medication use, and no frequent infections or hospitalizations. Anthropometric measurements showed significant short stature, with a height of 131 cm (<3rd percentile) and a weight of 34.7 kg (15th percentile). Growth chart records indicated that the patient's height had been consistently around the 3rd percentile since early childhood.

Testicular volume was measured between 8 mL and 10 mL, and pubic hair was at Tanner stage P2-P3 [4]. Coupled with an advanced bone age of 14 years, these findings strongly indicated early or rapidly progressing puberty, with a predicted adult height of 140-145 cm.

The patient currently holds a disability certificate. The parent has opted not to repeat formal intelligence quotient testing until the renewal of the certificate. According to the parents, a previous assessment suggested a cognitive delay of approximately two years relative to his chronological age.

Physical examination confirmed the orthopedic findings of contractures and fixed bilateral forearm pronation. No hepatosplenomegaly or corneal clouding was noted. In addition, the patient's characteristic facial dysmorphism was mild, mainly limited to a flat face and low-set ears, with only very mild brachydactyly observed in the hands.

**Figure 3:**
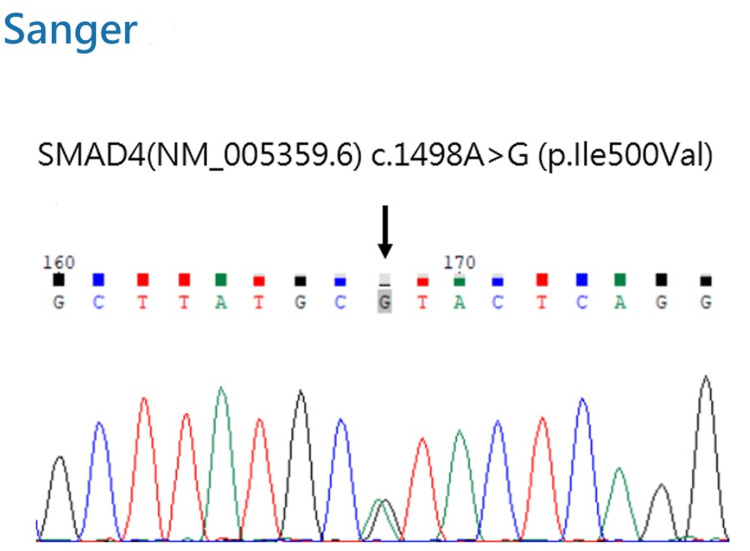
Sanger sequencing chromatogram of the SMAD4 gene The arrow indicates the heterozygous missense variant c.1498A>G (p.Ile500Val), confirming the diagnosis of Myhre syndrome.

**Figure 4:**
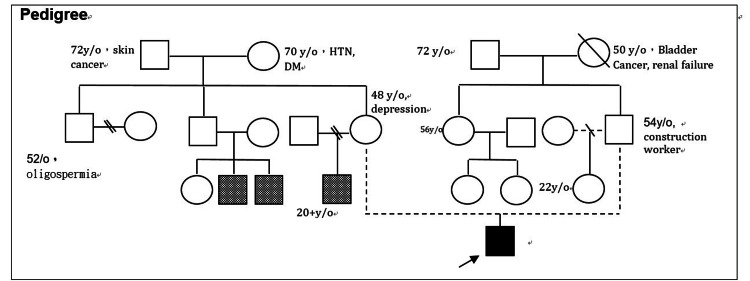
Three-generation family pedigree The pedigree illustrates the inheritance pattern and the clinical status of family members. Symbols follow standard pedigree nomenclature. HTN, hypertension; DM, diabetes mellitus

Although the clinical features were not entirely suggestive of MPS, a commercial genetic panel was utilized to systematically rule out metabolic storage diseases and address the initial referral suspicion. Notably, the analysis identified a known pathogenic heterozygous missense variant in the *SMAD4* gene: c.1498A>G (p.Ile500Val) [5], which was further confirmed using Sanger sequencing (Figure 3). Although the parents declined testing, a three-generation family pedigree was obtained (Figure 4).

Due to potential cardiopulmonary involvement in MS, an echocardiogram was performed, revealing coarctation of the aorta (CoA) (8 mm narrowed segment, continuous wave velocity: 2.47 m/s), mild mitral valve stenosis (MS), trivial mitral valve regurgitation (MR), and mild left pulmonary valve stenosis (PS). Notably, the patient's blood pressure remained within the normal range for his age despite the CoA finding.

Pulmonary function testing showed a maximal aerobic capacity of approximately 27% of the predicted value, corresponding to a functional aerobic impairment (FAI) of 73% (extreme FAI).

Based on the genetic findings and the presence of characteristic clinical features (Table 1), the patient was definitively diagnosed with MS.

**Table 1:** Clinical features of the present case in comparison with typical Myhre syndrome

Category	Phenotypes matching Myhre syndrome	Novel or rare manifestations
Growth	Short stature/prenatal growth deficiency [3]	
Head and neck	Low-set ears [1,3]	
Cardiovascular	Coarctation of the aorta [3]	Mitral valve regurgitation/mitral valve stenosis/pulmonary valve stenosis
Respiratory	Decreased pulmonary function test [6]	
Chest	Broad ribs [5]	
Skeletal	Decrease joint mobility/brachydactyly (mild) [3,5]	Proximal radioulnar synostosis/contractures of bilateral heel cords and rectus femoris muscles/tiptoe gait
Genitourinary	Precocious puberty [5]	
Neurology	Impaired intellectual development [1]	

Regarding the orthopedic findings, the patient's PRUS has remained stable, and the family has opted for conservative observation rather than further surgical intervention. As for cardiopulmonary involvement, the patient remains asymptomatic at present; however, the objective echocardiogram findings and impaired pulmonary function necessitate regular multidisciplinary follow-up.

## Discussion

MS (OMIM 139210) is a well-documented rare disorder with a pathogenesis linked to the transforming growth factor-β signaling pathway [2,7]. The specific heterozygous missense variant identified in our patient, SMAD4, c.1498A>G (p.Ile500Val), is a known pathogenic mutation previously described in the literature [2,5,8,9]. Furthermore, MS is an autosomal dominant inherited disorder that typically manifests as a sporadic case arising from a de novo mutation in the proband. Although the parents declined genetic testing, the inheritance pattern observed in our patient's pedigree is highly consistent with MS (Figure 4).

Musculoskeletal findings and novel features

MS is a multisystem disorder with diverse clinical manifestations that have been well-described [3,10]. Our patient exhibited several characteristic features, which are summarized in Table 1. The key musculoskeletal findings included PRUS, contractures of the bilateral heel cords and rectus femoris muscles, and a tiptoe gait. With the exception of PRUS, these findings align with the spectrum of decreased joint mobility typically reported. However, the presence of PRUS represents a distinct and novel feature in this case.

A review of the literature indicates that nonsyndromic cases with isolated PRUS are often associated with variants in genes such as *SMAD6* and *NOG*, or sex chromosome aneuploidies. A subgroup of patients with hematological abnormalities may present with *HOXA11* or *MECOM* gene variants [11]. Furthermore, PRUS is also found in complex congenital syndromes, including Pfeiffer, Poland, and Holt-Oram syndromes [11]. Therefore, the presence of PRUS in a syndromic patient warrants the inclusion of MS in the differential diagnosis.

Growth and sexual development

Short stature and prenatal growth deficiency are nearly universal features of MS [3,12]. Abnormalities in sexual development, including precocious puberty, cryptorchidism, and hypospadias, are also frequently reported in male patients [5]. At the age of 11 years and nine months, our patient was already at Tanner stage P2-3, with no other genital anomalies noted. Although baseline pubertal data were unavailable for a definitive comparison, relatively early-onset or rapid progressive puberty is likely. It is well-established that precocious puberty compromises final adult height by inducing premature fusion of the growth plates. Consequently, the co-occurrence of short stature and precocious puberty in MS patients is likely to result in a further reduction in their final adult height.

Cardiopulmonary involvement

The cardiovascular findings in our patient, notably CoA, align with previous reports. While some reports may not emphasize mitral valve stenosis, mitral valve regurgitation, and pulmonary stenosis, these likely represent the spectrum of valvular involvement in MS. Although the patient’s current clinical presentation is mild, some patients develop severe complications, such as cardiomyopathy, systemic hypertension, and pericarditis, eventually leading to fatal outcomes [12,13]. Therefore, lifelong cardiac monitoring is essential.

Pulmonary involvement in MS is well-documented. Structural features such as laryngotracheal stenosis [12], along with clinical symptoms including stridor, croup, frequent respiratory infections, chronic cough, and asthma, have been reported. Furthermore, sleep abnormalities are frequently observed [6]. Spirometry and oscillometry findings demonstrating central and peripheral obstruction, restriction, and air trapping are common in MS patients [6].

In our patient, the aforementioned clinical symptoms and diagnoses were absent. However, pulmonary function testing revealed extreme FAI (73%), which is consistent with the functional decline reported in the literature [6]. Furthermore, literature reports describe the combination of obstructive pathologies and recurrent pleural effusion leading to chronic respiratory failure [12]. Therefore, although our patient is currently asymptomatic, the existing pulmonary function data already demonstrate significant functional decline. Given these progressive risks, long-term follow-up and continuous monitoring are essential.

## Conclusions

MS presents a complex clinical spectrum, where PRUS may serve as a rare but significant skeletal manifestation. The identification of PRUS in association with the *SMAD4* c.1498A>G (p.Ile500Val) variant expands the known phenotypic range of this syndrome. Furthermore, the presence of abnormal echocardiographic findings and significant functional pulmonary impairment in an otherwise asymptomatic individual shows that cardiopulmonary involvement can be easily overlooked. Recognizing these multisystem involvements, supported by definitive genetic confirmation, facilitates a more comprehensive understanding of the disease's natural history and the importance of longitudinal clinical surveillance.
